# Relationship Between Thyroid Autoantibodies and Recurrence of Papillary Thyroid Carcinoma in Children and Adolescents

**DOI:** 10.3389/fonc.2022.883591

**Published:** 2022-06-08

**Authors:** Dongmei Huang, Jingtai Zhi, Jinming Zhang, Xuan Qin, Jingzhu Zhao, Xiangqian Zheng, Ming Gao

**Affiliations:** ^1^ Department of Thyroid and Neck Tumor, Tianjin Medical University Cancer Institute and Hospital, National Clinical Research Center for Cancer, Key Laboratory of Cancer Prevention and Therapy, Tianjin's Clinical Research Center for Cancer, Tianjin, China; ^2^ Department of Thyroid and Breast Surgery, Tianjin Union Medical Center, Tianjin, China; ^3^ Tianjin Key Laboratory of General Surgery Inconstruction, Tianjin Union Medical Center, Tianjin, China

**Keywords:** papillary thyroid carcinoma, children and adolescents, thyroid peroxidase antibody, thyroglobulin antibody, recurrence

## Abstract

**Background:**

Numerous studies reported connection between papillary thyroid carcinoma (PTC) and thyroid autoantibody in adults, but few of them have investigated whether there is a similar link in children and adolescents. The purpose of this research was to explore the relationship between clinicopathological features, prognosis and preoperative thyroid peroxidase antibody (TPOAb) as well as thyroglobulin antibody (TgAb) status in children and adolescents with PTC.

**Methods:**

This study retrospectively reviewed 179 patients of PTC who underwent a thyroidectomy from January 2000 to June 2021 at Tianjin Medical University Cancer Hospital. We compared preoperative TgAb and TPOAb status with the clinicopathological features and prognosis of children and adolescents with PTC in different age groups.

**Results:**

Patients with positive preoperative TPOAb and TgAb had lower recurrence rate in the younger group (*P* = 0.006, 0.047, respectively). Patients with positive TPOAb preoperatively had normal level of preoperative Tg and less cervical LNM than patients with negative TPOAb in children and adolescents (P < 0.05). Positive TPOAb preoperatively of PTC patients had a longer median DFS (113.4 months) than negative TPOAb (64.9 months) (P = 0.009, log-rank). Univariate analyses showed age, maximal tumor size, T stage, multifocality, lateral LNM and N staging were predictors for cancer recurrence in children and adolescents (P<0.05). Cox regression analysis found younger age (HR 0.224, P < 0.001), lateral LNM (HR 0.137, P = 0.010), N stage (HR 30.356, P < 0.001) were independent risk factors for recurrence.

**Conclusions:**

Our study found that presence of preoperative TPOAb and TgAb could serve as novel prognostic factors for predicting recurrence of PTC in children.

## Introduction

Thyroid carcinoma is less common in children and adolescents with around 0.5-3%, but the incidence has been steadily rising recently (1). Papillary thyroid carcinoma (PTC) is the most common type of thyroid carcinoma in both children and adults, with around 90% (2). The most common thyroid autoimmune disease among children with thyroiditis is Hashimoto’s thyroiditis (HT) (3), which is characterized by high titers of thyroid autoantibodies (4). Thyroid autoantibodies are usually present in the serum of HT patients, with 70-80% for thyroglobulin antibody (TgAb) and 90-95% for thyroid peroxidase antibody (TPOAb), considered to be sensitive markers for HT (5). TgAb and TPOAb positivity rate were significantly increasing in patients with PTC (6). The association between HT and PTC has been studied since the first report in 1955 by Dailey (7). However, the link between thyroid autoantibodies, clinicopathologic and prognosis remain equivocal. Paparodis et al. proposed high levels of TPOAb seemed to prevent PTC (8). Conversely, Adhami’ study (9) suggested positive TgAb was connected with lymph node metastases in PTC patients. Iliadou et al. (10) showed that thyroid carcinoma with HT presented more frequently invasive characteristics in children and adolescents ( ≤ 21years). The above findings demonstrated the inconsistent conclusions about connection between HT and PTC may be due to different antibody status, and the exact association is currently unclear. Thence, further researches on the effect of preoperative TPOAb and TgAb on the development and prognosis of PTC are needed.

Many studies have reported the relationship between HT and PTC in adults, but few researches have investigated whether there is a similar link in children and adolescents. Due to the low incidence of thyroid carcinoma, there have rarely retrospective studies of this age group in China that little is known about the clinical implication of preoperative thyroid autoantibodies. The study aimed to explore the connection between clinicopathological features, prognosis with preoperative TgAb and TPOAb status in children and adolescents with PTC. Relevant literature defined 21 as the age of separation between adults and adolescents (10, 11), which provides a basis for selecting and grouping of children and adolescents in this study.

## Methods

### Study Population

This study retrospectively reviewed 179 PTC patients who underwent initial thyroidectomy enrolled from January 2000 to June 2021 that carried out at Tianjin Medical University Cancer Hospital. All patients met the following criteria: (1) their age at diagnosis ≤ 21 years old; (2) histologically proven PTC after thyroidectomy; (3) thyroid-stimulating hormone (TSH), thyroglobulin (Tg), TPOAb and TgAb were measured before thyroidectomy. The exclusion criteria were the following: (1) they merged with other tumors; (2) they had serious medical record deficiency. The studies involving human participants were reviewed and approved by the Ethical Committee of the Tianjin Medical University Cancer Institute and Hospital. The patients provided written informed consent to participate in this study.

### Clinicopathological Variables

Patients’ characteristics such as age, gender, preoperative TPOAb, TgAb, TSH and Tg levels in the serum, postoperative histological type, pathological characteristics of maximal tumor size, bilaterality, multifocality, extrathyroidal extension (ETE), lymph nodes metastases (LNM) (N1a-central LNM, N1b-lateral LNM) were recorded. Serum TPOAb, TgAb, TSH and Tg were measured on a Roche Cobas immunology analyzer (Switzerland) using the electrochemiluminescence immunoassay (ECLIA) method. The normal ranges for serum levels of TPOAb, TgAb, TSH and Tg were 0–9 IU/mL, 0–4.1 IU/mL, 0.27–4.20 mlU/L, -1.4–78 ug/L, respectively. The intra-assay coefficient of variation (CV) values of serum TPOAb, TgAb, TSH and Tg were 2.4% to 5.6%, 1.3% to 4.9%, 3% to 4% and 1.6% to 4.1%, respectively, whereas the interassay CV values were 3.2% to 5.7%, 2.1% to 6.9%, 4% to 6% and 1.3% to 5.8%, respectively. TPOAb, TgAb, TSH and Tg were considered positive when their serum level was over the upper range. TNM staging was based on the 8th edition of the American Joint Committee on Cancer TNM staging system (12).

### Postoperative Follow-Up

The primary outcome was recurrence of disease. The primary imaging modality was ultrasonography during follow-up. When a suspected recurrent lesion (thyroid bed or lymphadenopathy) was identified by imaging and fine-needle aspiration cytology, then surgery was performed to remove the disease and confirm the diagnosis. Elevated serum Tg and TgAb levels without clinical evidence of structural disease were defined as isolated biochemical recurrence and were not classified as true recurrence (13, 14). Disease-free survival (DFS) was defined as the time interval from thyroidectomy to detect PTC recurrence. Follow-up for each patient could be recorded by reviewing records or by calling the patients.

### Statistical Analysis

Data analysis was performed by using SPSS v.26.0 (Chicago, IL, USA). Categorical variables were reported as absolute numbers and percentages, continuous variables were reported as a mean ± standard deviation or median and range. Differences between groups were assessed using the χ^2^ statistic and Fisher’s exact test (categorical variables) or the independent-samples t-test (continuous variables). The Kaplan-Meier method and log-rank test were used to analyze time-dependent variables. The Cox hazard regression model was used for multivariate analysis, expressed as hazard ratio (HR) with the 95%CI. A value of *P* < 0.05 was considered statistically significant.

## Results

### Study Populations

This study included preoperative TgAb and TPOAb data from 179 children and adolescents with PTC after thyroidectomy. The features of patients were given in **Table 1**. Patients consisted of 131 girls (73.2%) and 48 boys (26.8%). Thyroid involvement was multifocal in 97 patients (54.2%) and bilateral in 61 patients (34.1%). ETE was documented of 129 patients (72.1%). A total of 154 had central LNM (86.0%), 111 had lateral LNM (62.0%). During a mean follow-up of 74 months (2-225 months), 40 patients (22.3%) had a recurrence.

**Table 1:** Characteristics of the study patients.

Characteristics	N (%)	Characteristics	N (%)
**Gender**		**ETE**	
Female	131 (73.2)	Yes	129 (72.1)
Male	48 (26.8)	No	50 (27.9)
**Age at diagnosis**		**Preoperative TPOAb**	
<14 years	36 (20.1)	Positive	64 (35.8)
14-21 years	143 (79.9)	Negative	115 (64.2)
**Maximal tumor size**		**Preoperative TgAb**	
≤ 2cm	89 (49.7)	Positive	53 (29.6)
>2cm	90 (50.3)	Negative	126 (70.4)
**T staging**		**Preoperative TSH**	
T1a	18 (10.1)	Positive	30 (16.8)
T1b	70 (39.1)	Negative	149 (83.2)
T2	78 (43.6)	**Preoperative Tg**	
T3	13 (7.3)	Positive	56 (31.3)
**Central LNM**		Negative	123 (68.7)
Yes	154 (86.0)	**TNM staging**	
No	25 (14.0)	I	179 (100.0)
**Lateral LNM**		**Surgical approach**	
Yes	111 (62.0)	Total thyroidectomy	64 (35.8)
No	68 (38.0)	Sub-total thyroidectomy	25 (14.0)
**Cervical LNM**		Ipsilateral glandular lobe plus isthmus resection	90 (50.3)
Yes	162 (90.5)	**Lymph node dissection**	
No	17 (9.5)	Unilateral CLND	44 (24.6)
**N staging**		Unilateral MRND	62 (34.6)
N0	17 (9.5)	Unilateral MRND, plus contralateral CLND	19 (10.6)
N1a	46 (25.7)	Bilateral CLND	12 (6.7)
N1b	116 (64.8)	Bilateral MRND	42 (23.5)
**Bilaterality**		**RAI ablation**	
Yes	61 (34.1)	Yes	29 (16.2)
No	118 (65.9)	No	150 (83.8)
**Multifocality**		**Recurrence**	
Yes	97 (54.2)	Yes	40 (22.3)
No	82 (45.8)	No	139 (77.7)

LNM, lymph node metastases; ETE, extrathyroidal extension; TPOAb, thyroid peroxidase antibody; TgAb, thyroglobulin antibody; TSH, thyroid-stimulating hormone; Tg, thyroglobulin; TNM, tumour-node-metastasis; CLND, central lymph node dissection; MRND, modified radical neck dissection; RAI, radioactive iodine.

### Relationship Between Preoperative Thyroid Autoantibodies and Clinicopathologic Features of PTC in Different Age Groups

Divided patients into two groups according to relevant literature (15–17): the younger group (< 14 years old) and the older group (14-21 years old). We analyzed the relationship between TgAb, TPOAb and clinicopathological features of PTC patients.

We found that patients with positive preoperative TgAb were predominant female compared with negative TgAb patients in all patients and the older group, and preoperative Tg was usually within the normal range in all groups (*P* < 0.05). Moreover, the recurrence rate of positive preoperative TgAb patients was lower than negative TgAb patients in the younger group (*P* = 0.047) (**Table 2**).

**Table 2:** Clinicopathologic features of PTC patients with positive and negative TgAb in different age groups.

Variables	Total (n=179)			< 14 years (n=36)			14–21 years (n=143)		
	TgAb+	TgAb-	*P* value	TgAb+	TgAb-	*P* value	TgAb+	TgAb-	*P* value
**Gender (n, %)**									
female	45 (84.9)	86 (68.3)	0.022*	8 (61.5)	16 (69.6)	0.902	37 (92.5)	70 (68.0)	0.002*
male	8 (15.1)	40 (31.7)		5 (38.5)	7 (30.4)		3 (7.5)	33 (32.0	
**Preoperative Tg**									
positive	4 (7.5)	52 (41.3)	< 0.001*	1 (7.7)	12 (52.2)	0.021*	3 (7.5)	40 (38.8)	< 0.001*
negative	49 (92.5)	74 (58.7)		12 (92.3)	11 (47.8)		37 (92.5)	63 (61.2)	
**Maximal tumor size**									
≤ 2cm	27 (50.9)	62 (49.2)	0.832	5 (38.5)	6 (26.1)	0.691	22 (55.0)	56 (54.4)	0.946
>2cm	26 (49.1)	64 (50.8)		8 (61.5)	17 (73.9)		18 (45.0)	47 (45.6)	
**Multifocality**	27 (50.9)	70 (55.6)	0.572	10 (76.9)	17 (73.9)	1.000	17 (42.5)	53 (51.5)	0.336
**Bilaterality**	21 (39.6)	40 (31.7)	0.310	7 (53.8)	9 (39.1)	0.393	14 (35.0)	31 (30.1)	0.571
**ETE**	36 (67.9)	93 (73.8)	0.423	9 (69.2)	16 (69.6)	1.000	27 (67.5)	77 (74.8)	0.382
**Cervical LNM**	47 (88.7)	115 (91.3)	0.589	13 (100.0)	22 (95.7)	1.000	34 (85.0)	93 (90.3)	0.545
**Central LNM**	46 (86.8)	108 (85.7)	0.849	13 (100.0)	22 (95.7)	1.000	33 (82.5)	86 (83.5)	0.886
**Lateral LNM**	31 (58.5)	80 (63.5)	0.529	9 (69.2)	20 (87.0)	0.225	22 (55.0)	60 (58.3)	0.724
**RAI ablation**	7 (13.2)	22 (17.5)	0.481	2 (15.4)	8 (34.8)	0.389	5 (12.5)	14 (13.6)	0.863
**Recurrence**	9 (17.0)	31 (24.6)	0.264	4 (30.8)	15 (65.2)	0.047*	5 (12.50	16 (15.5)	0.645

TgAb, thyroglobulin antibody; Tg, thyroglobulin; ETE, extrathyroidal extension; LNM, lymph node metastases; TNM, tumour-node-metastasis; RAI, radioactive iodine; *P < 0.05.

As shown in **Table 3**, our findings suggested that positive preoperative TPOAb patients had normal level of preoperative Tg and less cervical LNM than patients with negative TPOAb in all patients and the older group (*P* < 0.05). In addition, patients with positive TPOAb had lower recurrence rate in the younger group (*P* = 0.006).

**Table 3:** Clinicopathologic features of PTC patients with positive and negative TPOAb in different age groups.

Variables	Total (n=179)			< 14 years (n=36)			14–21 years (n=143)		
	TPOAb+	TPOAb-	*P* value	TPOAb+	TPOAb-	*P* value	TPOAb+	TPOAb-	*P* value
**Gender (n, %)**									
female	51 (79.7)	80 (69.6)	0.143	7 (63.6)	17 (68.0)	1.000	44 (83.0)	63 (70.0)	0.083
male	13 (20.3)	35 (30.4)		4 (36.4)	8 (32.0)		9 (17.0)	27 (30.0)	
**Preoperative Tg**									
positive	9 (14.1)	47 (40.9)	< 0.001*	1 (9.10	12 (48.0)	0.063	8 (15.1)	35 (38.9)	0.003*
negative	55 (85.9)	68 (59.1)		10 (90.0)	13 (52.0)		45 (84.9)	55 (61.1)	
**Maximal tumor size**									
≤ 2cm	29 (45.3)	60 (52.2)	0.379	4 (36.4)	7 (28.0)	0.913	25 (47.2)	53 (58.9)	0.147
>2cm	35 (54.7)	55 (47.8)		7 (63.6)	18 (72.0)		28 (52.8)	37 (41.1)	
**Multifocality**	34 (53.1)	63 (54.8)	0.831	9 (81.8)	18 (72.0)	0.835	25 (47.2)	45 (50.0)	0.744
**Bilaterality**	25 (39.1)	36 (31.3)	0.294	7 (63.6)	9 (36.0)	0.241	18 (34.0)	27 (30.0)	0.622
**ETE**	44 (68.8)	85 (73.9)	0.461	8 (72.7)	17 (68.0)	1.000	36 (67.9)	68 (75.6)	0.322
**Cervical LNM**	54 (84.4)	108 (93.9)	0.037*	11 (100.0)	24 (96.0)	1.000	43 (81.1)	84 (93.3)	0.025*
**Central LNM**	51 (79.7)	103 (89.6)	0.068	11 (100.0)	24 (96.0)	1.000	40 (75.5)	79 (87.8)	0.057
**Lateral LNM**	36 (56.3)	75 (65.2)	0.236	7 (63.6)	22 (88.0)	0.167	29 (54.7)	53 (58.9)	0.626
**RAI ablation**	6 (9.4)	23 (20.0)	0.064	1 (9.1)	9 (36.0)	0.209	5 (9.4)	14 (15.6)	0.298
**Recurrence**	10 (14.3)	30 (26.1)	0.107	2 (18.2)	17 (68.0)	0.006*	8 (15.1)	13 (14.4)	0.916

TPOAb, thyroid peroxidase antibody; Tg, thyroglobulin; ETE, extrathyroidal extension; LNM, lymph node metastases; TNM, tumour-node-metastasis; RAI, radioactive iodine; * P < 0.05.

### Multivariate Analysis for Variables Associated With PTC Recurrence

**Figure 1:**
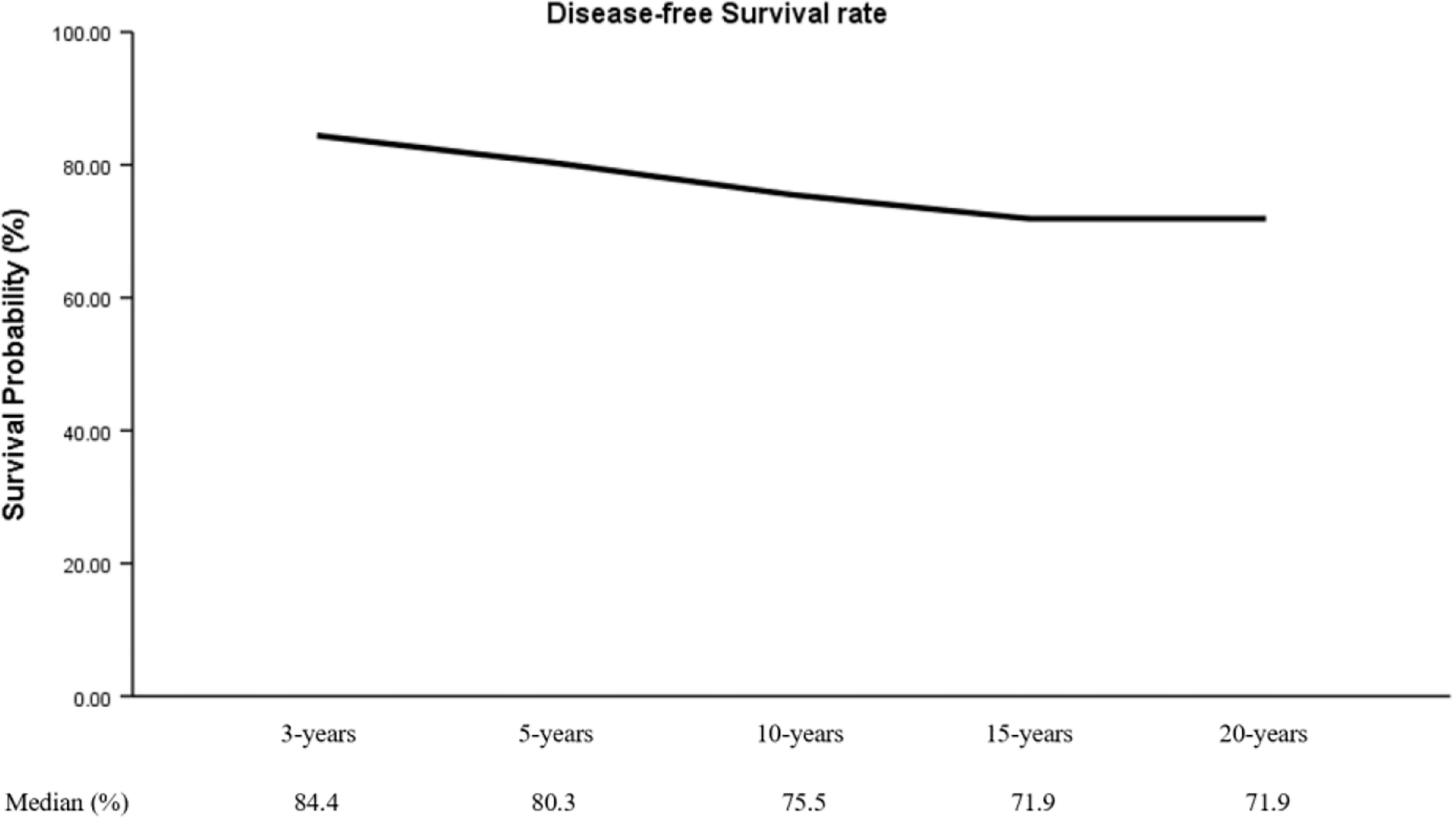
Tendency chart of pooled DFS rate.

During a mean follow-up of 74 months (2-225 months), 40 patients (22.3%) had a recurrence: 31 (77.5%) were negative preoperative TgAb and 30 (75.0%) were negative preoperative TPOAb. Moreover, this study showed that DFS rate at 3 years (DFSR-3y), DFSR-5y, -10y, -15y, and -20y were 84.4%, 80.3%, 75.5%, 71.9%, and 71.9%, respectively (**Figure 1**).

**Figure 2:**
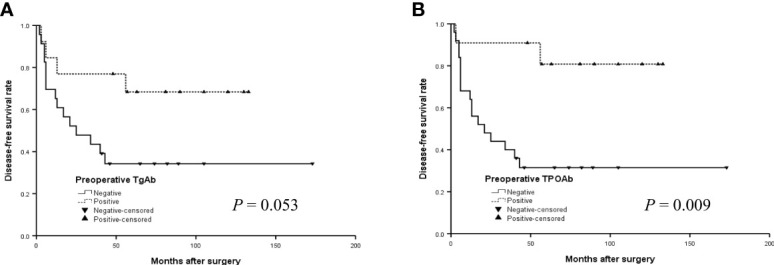
The disease-free survival (DFS) curves of the positive or negative preoperative TgAb **(A)** and TPOAb **(B)** in the younger group (< 14 years) with PTC.

The survival curves of DFS of preoperative TgAb and TPOAb status in the younger group were shown in **Figure 1**. The median DFS was 97.4 months for patients with positive preoperative TgAb and 69.8 months for patients with negative TgAb (**Figure 2A**) (*P* = 0.053, log-rank). Patients with positive TPOAb had longer median DFS (113.4 months) than negative TPOAb patients (64.9 months) (**Figures 2B**) (*P* = 0.009, log-rank).

**Figure 3:**
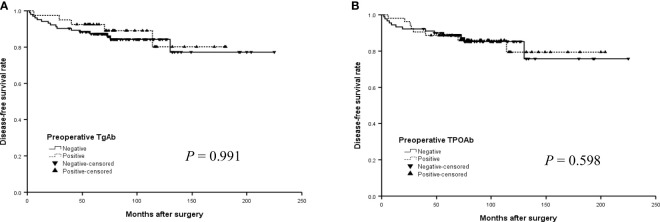
The disease-free survival (DFS) curves of the positive or negative preoperative TgAb **(A)** and TPOAb **(B)** in the older group (14 -21 years) with PTC.

**Figure 4:**
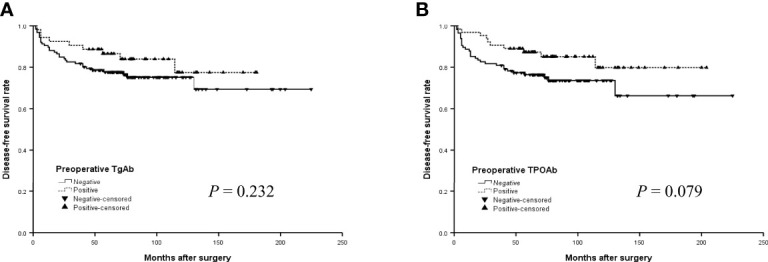
The disease-free survival (DFS) curves of the positive or negative preoperative TgAb **(A)** and TPOAb **(B)** in all patients with PTC.

The median DFS did not differ significantly by TgAb and TPOAb status in the older group (**Figures 3A**, **B**) (*P*>0.05, log-rank). In addition, the median DFS was 152.4 months for positive TgAb patients and 169.6 months for those negative TgAb, 173.7 months for positive TPOAb patients and 164.7 months for those negative TPOAb. The differences were also not significant in all patients with PTC (**Figures 4A**, **B**) (*P*>0.05, log-rank).

After adjusting for other clinicopathological factors (age, gender, cervical LNM, multifocality, T stage, N stage, maximal tumor size, etc.), we summarized the outcomes of multivariate analysis of the association of preoperative TgAb and TPOAb with cancer recurrence. The results in **Table 4** showed risk factors (age, maximal tumor size, T stage, multifocality, lateral LNM, N stage, preoperative TSH and Tg level) were predictors for cancer recurrence in children and adolescents (P < 0.05). Moreover, preoperative positive TPOAb was associated with better prognosis in the younger group (*P* = 0.021). Cox regression analysis found that younger age (HR 0.224, *P* < 0.001), lateral LNM (HR 0.137, *P* = 0.010), N stage (HR 30.356, *P* < 0.001) were independent risk factors for recurrence of PTC in children and adolescents.

**Table 4:** Cox proportional hazard regression analysis for variables associated with PTC recurrence at different ages.

Variables	Univariate			Multivariate		
	HR	95%CI	*P* value	HR	95%CI	*P* value
**<14 years old group**						
Positive preoperative TPOAb	0.176	0.040-0.771	0.021*			Ns
Positive preoperative TgAb	0.355	0.117-1.078	0.068			Ns
**14-21years old group**						
Positive preoperative TPOAb	0.995	0.412-2.405	0.991			Ns
Positive preoperative TgAb	0.764	0.280-2.086	0.599			Ns
**All patients**						
The younger age	0.198	0.106-0.37	< 0.001*	0.224	0.110-0.455	< 0.001*
Positive preoperative TPOAb	0.533	0.260-1.091	0.085			Ns
Positive preoperative TgAb	0.639	0.304-1.343	0.237			Ns
Positive preoperative TSH	2.024	1.008-4.064	0.047*			Ns
Positive preoperative Tg	1.941	1.04-3.623	0.037*			Ns
Maximal tumor size>2cm	2.883	1.438-5.782	0.003*			Ns
T stage	1.659	1.105-2.490	0.015*			Ns
Multifocality	2.271	1.154-4.469	0.018*			Ns
Lateral LNM	3.258	1.440-7.370	0.005*	0.137	0.030-0.622	0.010*
N stage	6.264	2.058-19.062	0.001*	30.356	6.044-152.479	< 0.001*

TPOAb, thyroid peroxidase antibody; TgAb, thyroglobulin antibody; TSH, thyroid-stimulating hormone; Tg, thyroglobulin; LNM, lymph node metastases; *P < 0.05.

## Discussion

The National Cancer Institute’s Surveillance, Epidemiology and End Results (SEER) results (2) showed that approximately 90% of thyroid carcinoma pathological types were PTC in children and adolescents, consistent with adults. However, children and adolescents with PTC have their own unique biological characteristics (18), being highly aggressive, prone to metastases and high recurrence rate. Therefore, it is necessary to further study the clinicopathological features of this age group to provide a basis for clinical diagnosis and treatment. The Adham’s and Shan’s studies have shown thyroid autoantibody were significantly associated with development and prognosis of PTC (9, 19). However, different autoantibody status may have different effects on PTC, and the relationship between PTC and autoantibodies in children and adolescents is still debated (20, 21). Thence, this study was aimed to investigate whether preoperative autoantibody can predict clinicopathologic features and prognosis of PTC in children and adolescents.

Association between thyroid autoantibodies and clinicopathologic features, such as tumor size, bilaterality, multifocality, ETE, and LNM still remain equivocal. Song’ study showed PTC patients with positive TgAb and TPOAb had better clinicopathologic features (22). But Li et al. suggested the presence of antibodies did not affect the tumor size (19). Consistent with previous studies that there was no difference in ETE, multifocality between different TgAb and TPOAb status of PTC patients (23, 24), our findings suggested both preoperative TgAb and TPOAb were not associated with multifocality, bilaterality, and ETE of PTC patients. We analyzed the differences in clinicopathological characteristics of different autoantibody status in different age groups, which showed preoperative positive TgAb and TPOAb have been associated with better prognosis in younger children (*P* < 0.05). In the older group, preoperative positive TgAb patients were more female, and preoperative Tg was usually within the normal range compared to negative TgAb patients (*P* < 0.05). Preoperative positive TPOAb patients often had normal preoperative Tg level and less cervical LNM compared to negative TPOAb patients (*P* < 0.05). In this study, 45 patients presented both TgAb and TPOAb. We further analyzed preoperative Tg levels in four antibody status and found that there were differences between groups, which suggested that the observation of Tg levels in different TPOAb status may be affected by TgAb status. Therefore, it is necessary to measure TPOAb and TgAb levels preoperatively simultaneously.

Cervical LNM is known to be a key predictor of recurrence in PTC patients (25). However, association between thyroid autoantibodies and cervical LNM in PTC patients is still controversial. Jo’s study (23) indicated that positive TgAb patients had a significantly increased risk of cervical LNM (*P* = 0.010). In contrast, other researchers reported no difference in LNM between positive and negative TgAb patients with PTC (20). On the other hand, Li et al. (19) pointed positive TPOAb reduced the risk of cervical LNM in patients with PTC. But Lee et al. (24) grouped 1879 patients with PTC based on the presence of TPOAb, and found no difference in LNM between positive and negative TPOAb groups. However, we analyzed preoperative TgAb and TPOAb levels of 179 PTC patients and found that patients with preoperative positive TPOAb had less LNM in children and adolescents (*P* < 0.05). Therefore, the effect of preoperative TgAb and TPOAb on LNM in children and adolescents with PTC still needs to include more cases for further research.

Our study showed that thyroid autoantibodies were associated with better clinicopathologic features in children and adolescents with PTC. The possible potential mechanism was that TgAb could specifically recognize epitopes on the Tg of PTC patients to cause tumor epitope-specific immune responses, increase the destruction of cancer cells and exert its protective effect by regulating the tumor microenvironment (26, 27). TPOAb may mediate by complement-mediated cell death and/or antibody-dependent cytotoxicity to exert its anti-cancer effect (28). Positive TgAb and TPOAb were associated with an increasing number of tumor-associated lymphocyte proliferating cells, which showing better primary tumor characteristics and disease-free survival in children and adolescents with PTC (29). The study has certain clinical value, but unfortunately, the relevant mechanism was not found in this paper. Therefore, the potential mechanism between preoperative TgAb, TPOAb and PTC in children and adolescents needs further research.

PTC in children and adolescents is less common, but the recurrence rate is high (16.7-31.6%) (30–32). The results of our study showed that 40 patients (22.3%) had a recurrence, which was similar to Rubinstein’s study (33). Currently, the impact of preoperative TgAb and TPOAb on the prognosis of PTC patients remains controversial (22, 34, 35). Song et al. (20) revealed patients with positive TPOAb were associated with better DFS. But Durante et al. (36) evaluated 1,240 patients from 10 hospitals and indicated recurrence was more common in positive TgAb patients. McLeod et al. (37) showed TgAb status was not associated with DFS or overall survival. However, the aforementioned studies consisted of data from postoperative patients. Therefore, it is necessary to research the relationship between preoperative TgAb as well as TPOAb and prognosis in patients with PTC. Our study found preoperative positive TgAb and TPOAb were protective factor for recurrence in younger group (*P* < 0.05). But the results of univariate analyses showed that cancer recurrence were not associated with TgAb and TPOAb status in the older group (*P*>0.05). Therefore, more cases need to be included for further research.

Preoperative TPOAb and TgAb levels in the serum were relatively stable. Our study showed patients with preoperative positive TgAb and TPOAb had lower recurrence rate in the younger group of PTC patients, which was similar to other research findings (20, 38). Some studies suggested that postoperative TgAb levels may predict recurrence (36, 39), but postoperative antibodies were easily affected by other factors (which may be because cervical lymph nodes initiate and disseminate the autoimmune response or reflect the persistence of Tg in antigen-presenting cells (40, 41)), which may lead to inaccurate results. In some patients with positive TgAb preoperatively, the postoperative antibody level will decline over time, and it will turn negative after about 3 years (42, 43). Some studies showed that TgAb may persist for years after thyroidectomy, without clear evidence of persistent disease (40, 41). Therefore, it is necessary to further study the preoperative TgAb level and regularly measure TgAb postoperatively to evaluate the prognostic value of the change of postoperative TgAb in PTC patients with preoperative positive TgAb.

There have some limitations in this study. The retrospective study was conducted in a single center that might limit the general applicability of our findings. Therefore, further large-sample studies are needed to evaluate the effect of thyroid autoantibodies on LNM and recurrence.

## Conclusions

In conclusion, we found that presence of preoperative TPOAb and TgAb could serve as novel prognostic factors for predicting recurrence of PTC in children. Further studies are needed to measure TPOAb and TgAb periodically to confirm the prognostic value of postoperative changes in TPOAb and TgAb with positive preoperative TPOAb and TgAb in PTC patients.

## Data Availability Statement

The original contributions presented in the study are included in the article/supplementary material. Further inquiries can be directed to the corresponding authors.

## Ethics Statement

The studies involving human participants were reviewed and approved by the Ethical Committee of the Tianjin Medical University Cancer Institute and Hospital. Written informed consent to participate in this study was provided by the participants’ legal guardian/next of kin.

## Author Contributions

DH: Conceptualization, data collection and analysis, methodology, and drafting the manuscript. JTZ, JMZ: Conceptualization, data collection and analysis, and methodology. XQ, JZZ: Conceptualization and methodology. XZ, MG: Conceptualization, and manuscript review and editing. All authors contributed to the article and approved the submitted version.

## Funding

This work was supported by grants from the National Natural Science Foundation of China (81872169, 82172821, 82103386), Tianjin Municipal Science and Technology Project (19JCYBJC27400, 21JCZDJC00360) and Beijing-Tianjin-Hebei Basic Research Cooperation Project (20JCZXJC00120), The Science & Technology Development Fund of Tianjin Education Commission for Higher Education (2021ZD033), Tianjin Medical Key Discipline (Specialty) Construction Project.

## Conflict of Interest

The authors declare that the research was conducted in the absence of any commercial or financial relationships that could be construed as a potential conflict of interest.

## Publisher’s Note

All claims expressed in this article are solely those of the authors and do not necessarily represent those of their affiliated organizations, or those of the publisher, the editors and the reviewers. Any product that may be evaluated in this article, or claim that may be made by its manufacturer, is not guaranteed or endorsed by the publisher.

## Abbreviations

PTC, papillary thyroid carcinoma; TPOAb, thyroid peroxidase antibody; TgAb, thyroglobulin antibody; TSH, thyroid-stimulating hormone; Tg, thyroglobulin; LNM, lymph node metastases; ETE, extrathyroidal extension; TNM, tumour-node-metastasis; DFS, disease-free survival; HR, hazard ratio; CI, confidence interval; CV, coefficient of variation.
